# Fit-for-Use Nanofibrillated Cellulose from Recovered Paper

**DOI:** 10.3390/nano13182536

**Published:** 2023-09-11

**Authors:** Ana Balea, M. Concepcion Monte, Elena Fuente, Jose Luis Sanchez-Salvador, Quim Tarrés, Pere Mutjé, Marc Delgado-Aguilar, Carlos Negro

**Affiliations:** 1 Department of Chemical Engineering and Materials, University Complutense of Madrid, Avda Complutense s/n, 28040 Madrid, Spainhelenafg@ucm.es (E.F.);; 2 LEPAMAP Research Group, University of Girona, Maria Aurèlia Capmany, 6, 17003 Girona, Spainpere.mutje@udg.edu (P.M.); m.delgado@udg.edu (M.D.-A.)

**Keywords:** recycled fibers, nanocellulose, enzymatic pretreatments, TEMPO-mediated oxidation, refining, high-pressure homogenization

## Abstract

The cost-effective implementation of nanofibrillated cellulose (CNF) at industrial scale requires optimizing the quality of the nanofibers according to their final application. Therefore, a portfolio of CNFs with different qualities is necessary, as well as further knowledge about how to obtain each of the main qualities. This paper presents the influence of various production techniques on the morphological characteristics and properties of CNFs produced from a mixture of recycled fibers. Five different pretreatments have been investigated: a mechanical pretreatment (PFI refining), two enzymatic hydrolysis strategies, and TEMPO-mediated oxidation under two different NaClO concentrations. For each pretreatment, five high-pressure homogenization (HPH) conditions have been considered. Our results show that the pretreatment determines the yield and the potential of HPH to enhance fibrillation and, therefore, the final CNF properties. These results enable one to select the most effective production method with the highest yield of produced CNFs from recovered paper for the desired CNF quality in diverse applications.

## 1. Introduction

Papermaking is one of the most sustainable industries contributing to the circular economy [1]. Recovered paper is widely recognized as an efficient and eco-friendly cellulose source for paper and board production. In Europe, 55.9% of raw materials come from recovered paper, which corresponds to a paper recycling rate of 70.5% [2]. In Spain, these values are even higher (92% and 71%, respectively). The use of recovered paper as raw material presents several challenges, due to the relatively low quality of the secondary fibers after several recycling cycles, which mainly affect the mechanical properties of the recycled paper due to shortening and hornification of the fibers during each recycling process. To enhance the quality of the recycled fibers and achieve the desired properties in the final product, papermakers use fiber refining and strengthening additives. In this context, extensive research has been carried out in the last decade to explore the potential of nanocellulose-based products as paper-reinforced additives. A lot of different nanomaterials have been development during the last three decades, such as carbon nanotubes, 2D mono-elemental graphene-like materials, and 2D β-Indium sulfide nanoplates [3,4]. Some of these have been combined with nanocellulose to improve paper properties or to develop new materials [5]. It is well known that CNFs improve interfiber bonding, significantly increasing the mechanical properties of the recycled products [6]. However, the use of CNF is still limited at industrial scale. This is mainly due to the high cost of nanocellulose products and to some limits in their application mainly due to their effect on drainage and the dispersion degree of the 3D network as has been recently demonstrated [7]. To minimize these limitations, new approaches are being developed based on the in situ production and use of fit-for-use CNFs.

Although CNFs can be obtained from a wide variety of cellulose sources such as wood (hardwood and softwood), seed fibers (cotton, coir, etc.), bast fibers (flax, hemp, jute, kenaf, ramie, etc.), grasses (bagasse, bamboo, etc.), marine animals (tunicate), algae, fungi, invertebrates, and bacteria [8], the trend for in situ production is focused on the utilization of virgin and secondary pulps, depending on the plant location [6]. Although virgin pulps have been widely studied for CNF production at industrial scale, further knowledge related to the use of recycled pulp is still necessary.

The methods used to obtain CNFs include a variety of fabrication techniques, each of which is categorized using different labels like top-down and bottom-up approaches, or according to their nature, which includes physical, chemical, and biological methods, or even depending on whether they involve spinning or non-spinning techniques [9]. Spinning methods can be further categorized into electrospinning techniques, which utilize electric voltage for fiber morphology control; and alternative spinning approaches which employ forces like pressurized air (bubble electrospinning) or centrifugal forces (centrifugal spinning) [10]. Among all of them, physical methods (top-down approaches) based on intensive mechanical pressure (high-pressure homogenization, microfluidization, grinding, refining, or milling) are commonly used to obtain CNFs from virgin and secondary cellulose fibers. Prior to this mechanical treatment, various pretreatments, including chemical, enzymatic, mechanical, or combined approaches, can be employed to enhance nanofibrillation and the separation of the individual nanofibers. Furthermore, the pretreatments contribute to reduce the energy demand of the subsequent mechanical treatment [11].

On the other hand, the final properties of the CNFs are influenced by several factors, including the cellulose raw material used, the type and intensity of the pretreatment, and the type and severity of the mechanical defibrillation process. These variables collectively determine the final characteristics of the produced CNFs [12]. Different properties are required based on the final application. For example, the medical and electronic fields require CNFs with high purity, fibrillation yield, and homogeneity [13,14] and transparent nanopaper production requires high transmittance [15], but these properties are not always key for reinforcing some composites or paper products [6].

There are several studies related to the use of recovered paper to obtain CNFs at lab scale. Some researchers have employed different types of recycled cellulosic materials, such as recycled newspapers [16]; recycled pulp [17]; waste paper [18,19]; old corrugated boards [20,21] or recycled milk-container board [22]. In these studies, CNFs were obtained through ultrafine grinding or sonication as mechanical treatments, without the application of any pretreatment. Only Ukkola et al. (2021) pretreated the pulps in a deep eutectic solvent solution based on choline chloride and urea to obtain nanofoams from crosslinked CNFs [22]. Recent studies have focused on utilizing deinking pulp (DIP) as raw material to obtain CNFs. Le Van et al. (2018) successfully obtained CNFs by employing TEMPO-mediated oxidation followed by homogenization in a digital homogenizer mixer [23]. Ang et al. (2020 and 2021) produced CNFs through a combination of mechanical pretreatment (PFI refining) and high-pressure homogenization (HPH) [24,25]. Zambrano et al. (2021) obtained CNFs solely through ultrafine grinding using DIP as raw material [26]. Balea et al. (2019) produced CNFs from two different types of recycled pulps, old newsprint (ONP) and old corrugated container (OCC), at different TEMPO-mediated oxidation levels prior to the homogenization process [6]. The state of the art shows a fragmentated knowledge from which is not possible to produce fit-for-use CNFs.

The effect of different pretreatments and homogenization conditions have been studied for virgin bleached softwood and hardwood chemical pulps [27] and thermomechanical pulp [28] which also determined the energy applied for the nanofibrillation process. However, to the best of our knowledge, the impact of treatment intensity, involving various pretreatment methods and homogenization conditions applied to recovered paperboard, on the properties of the resulting CNFs and the associated energy consumption remains unexplored. 

Recently, Ang et al. (2021) produced several CNF nanopapers from DIP using different combinations of mechanical refining (10,000, 30,000, and 50,000 revolutions in PFI) and HPH at 1000 bar (1 pass and 3 passes) [24]. They found that the nanofibrillation efficiency for recycled fibers was lower than that for virgin bleached eucalyptus kraft pulp due to hornification during papermaking. Therefore, results from virgin pulps cannot be transferred to recycled pulp, since the pulp composition is different, as paperboard contains more lignin and ashes, and the fibers are hornified due to the drying process. Therefore, recycled pulps will behave differently during CNF production. 

In terms of energy consumption, Josset et al. (2014) conducted an energy-related study on the direct fibrillation of recycled newspaper and wheat straw through a grinding process [16]. They compared the CNFs obtained from these raw materials with CNFs derived from a bleached wood pulp (ECF) by evaluating the mechanical properties and specific surface areas of the fibrillated materials. The study found that the energy inputs required to achieve optimal mechanical properties in the prepared films from recycled newspaper were higher compared to the ECF, ranging from approximately 4 to 5 kWh/kg. Another study by Ozola et al. (2019) estimated that the production of CNFs from recycled pulp, without deinking, had higher specific energy consumption (0.865 kWh/kg waste) compared to the production of other products such as egg packaging or cardboard [29]. Overall, these studies demonstrate that the production of CNFs from different types of recycled papers is feasible in terms of improving the quality of the final product. However, it is important to consider the energy consumption associated with the specific production processes for effective resource management and sustainability.

This research aims to generate new knowledge on the effects of different treatment combinations on the CNF properties obtained from recovered paperboard. Five different pretreatments are considered, including a mechanical pretreatment through PFI refining (20,000 revolutions), two enzymatic hydrolysis approaches (80 mg/kg and 240 mg/kg), and TEMPO-mediated oxidation under two different conditions (5 mol NaClO/kg and 15 mol NaClO/kg). The study explores five different conditions for the HPH mechanical process for each pretreatment method. Furthermore, the energy requirements associated with the different treatment processes are assessed. This information will facilitate the selection of a fibrillation process based on the required CNF properties. 

## 2. Materials and Methods

### 2.1. Materials

OCC was selected as the recycled cellulose source as it is one of the most significant types of recovered papers for recycling, and it is listed in the European List of Standard Grades of Recovered Paper and Board (EN 643) as Group 1 in ordinary qualities (1.05). Liner and fluting, used in proportions of 35 and 65% for OCC pulp preparation, were kindly provided by SAICA (Zaragoza, Spain). This fluting/liner ratio is the most common for cardboard boxes. The chemical composition of the recycled pulp is presented in Table 1. A commercial monocomponent enzyme cocktail, Novozym 476, was kindly supplied by Novozymes A/S (Bagsværd, Denmark). This enzyme cocktail contains 2% (*w*/*v*) of endo-β-1,4-glucanases, with an enzyme activity of 341 U/mL. The other reagents utilized in this research were purchased from Sigma Aldrich (Madrid, Spain).

### 2.2. Cellulose Nanofiber Production

OCC pulp was prepared from a mixture of 35% liner and 65% fluting OCC paper by disintegration at 3% at 3000 rpm for 20 min using a standardized 2 L lab pulper. The resulting pulp was then adjusted to the desired pretreatment consistency by diluting it with distillated water or filtering it through a qualitative paper filter using a Buchner funnel. The production of CNFs involves a two-step process, beginning with a pretreatment stage followed by mechanical defibrillation using HPH. Different pretreatments were explored, and five levels of HPH energy were applied during the process.

#### 2.2.1. Pretreatments

The five different pretreatments considered in the study are summarized in Table 2. 

The mechanical pretreatment was conducted in accordance with the ISO standard 5264-2 (2011) [30]; the enzymatic hydrolysis procedure followed the methodology outlined in Tarrés et al. (2016) [31] and TEMPO-mediated oxidation was performed following the method described by Saito et al. (2007) [32]. 

#### 2.2.2. High-Pressure Homogenization

In this research, a total of 25 different types of CNFs were produced. The nanofibrillation of the samples was carried out by passing the pretreated pulp suspensions at 1 % of consistency through a PANDA Plus 2000 laboratory homogenizer (Gea Niro Soavi, Italy) using different pressure sequences as detailed in Table 3. 

All CNFs produced were named using the following nomenclature: Pretreatment_dose-CNF-HPH sequence number. As an example of this, CNFs produced through enzymatic hydrolysis pretreatment using an enzyme dosage of 80 mg/kg, followed by a HPH sequence of three passes at 300 bar, three passes at 600 bar, and three passes at 900 bar, are designated Enz_80-CNF-5.

The energy consumption of the HPH process was continuously monitored using a Circutor CVM-C10 measuring device (Barcelona, Spain), providing real-time readings of the consumed energy. The energy consumption was recorded after every HPH cycle and was then normalized to the processed dry mass.

### 2.3. Characterization of the Pulps

#### 2.3.1. Chemical Composition

The composition analysis of both unpretreated and pretreated pulps (Table 4) was conducted according to TAPPI T204 for extractive determination, TAPPI T211 for ash determination, and NREL/TP-510-42618 for additional laboratory analytical procedures [33,34,35]. Briefly, the extractives were determined using the Soxhlet extraction method with acetone as solvent. The ash content was determined through calcination at 525 °C. For lignin analysis, the following steps were followed: (i) acid hydrolysis of 0.3 g of dry pulp using 3 mL of 72 % H_2_SO_4_ for 1 h at 30 °C, (ii) dilution with 84 g of distilled water to prevent phase separation between high and low concentration acid layers, and (iii) autoclaving at 121 °C for 1 h, followed by filtration to determine Klason lignin by drying and weighing the filtration cake. The percentage of soluble lignin, cellulose, and hemicellulose were determined by analyzing the filtrate; soluble lignin was obtained by measuring the absorbance of the filtrate at 240 nm using a UV–vis spectrophotometer. Cellulose and hemicellulose were analyzed using high-performance liquid chromatography (HPLC) of the filtrate after neutralization with CaCO_3_ and microfiltration through a 0.2 µm filter.

The behavior of pretreated pulps is influenced by the functional groups present on the surface of cellulose. Therefore, conductimetric titration was used to determine the content of carboxyl groups per gram of the pretreated pulps, by following the method described in previous studies. In this method, a dried sample weighing between 0.05 and 0.1 g was suspended in 15 mL of 0.01 N HCl solution and stirred for 10 min and then taken to a conductivity sensor. The titration was performed by consecutively adding 0.1 mL of 0.05 N NaOH solution to the suspension until a noticeable increase in conductivity was observed. As the alkali neutralizes the chlorohydric acid, the conductivity initially decreases. Once the acid groups are neutralized, the conductivity remains constant. Finally, the excess of NaOH leads to an increase in conductivity. The number of carboxyl groups was calculated from the titration curve, which relates conductivity vs. amount (meq) of NaOH added, following the methodology described by Habibi et al. (2006) [36].

#### 2.3.2. Morphology

The morphological analysis encompassed several parameters, including average length weighted in length (length_w_), diameter (d), coarseness, and fine percentage (%). These measurements were determined using image analysis performed with a MorFi Compact analyzer (TechPap, Gières, France). However, as the resolution of MorFi images was limited and we were unable to accurately measure nanofibers, the aspect ratio of the pretreated samples was estimated using the measurement of the gel point (GP) based on the methodology developed by Varanasi et al. (2013) and Sanchez-Salvador et al. (2021) [37,38]. 

Furthermore, optical images of the pretreated pulps were taken using a Zeiss Axio Lab.A1 optical microscope and a color microscope camera Zeiss AxioCam ERc 5s (Carl Zeiss Microscopy GmbH, Göttingen, Germany). Subsequently, the optical images were processed using the version 1.53i of ImageJ software package to enhance the clarity of the images for further analysis.

### 2.4. Characterization of the CNFs

Characterization of the CNFs involved the measurement of several properties, including nanofibrillation yield, transmittance at 600 nm, cationic demand (CD), and aspect ratio of the CNF suspensions, following the methods described in previous studies [6,27]. To determine the nanofibrillation yield, CNF suspension was diluted to 0.1 wt.% and centrifugated at 4500× *g* for 30 min to isolate nanofibrillated cellulose from non-nanofibrillated components, which settled in the sediment. The weight of the sediment was measured to calculate the nanofibrillation yield. A UV–vis Shimadzu spectrophotometer UV-160A was used to measure the transmittance of the CNF suspensions at two different wavelengths. The anionic character of CNFs correlates with their CD, which was determined via back titration, with polydiallyldimethylammonium chloride (PDADMAC) and polyvinylsulfate potassium salt using a Mütek PCD04 particle charge detector (BTG Instruments, Weßling, Germany). The aspect ratio of the CNFs was determined using a recently developed methodology by Sanchez-Salvador et al. (2021) [38]. This approach modified the traditional GP methodology by dying the fibers with crystal violet, enabling the visualization of the fibril sedimentation and optimizing the sedimentation time to ensure complete settling. 

An optical microscope was used to obtain images from CNF hydrogels for qualitative support of our results. In this case, the parameters and method used for obtaining these images were consistent with those utilized for the pretreated pulps. Additionally, the optical images underwent processing with the ImageJ software package to seamlessly eliminate the continuous background. This was achieved by applying the “Process › Subtract Background” command from the menu, with the specified parameters including a rolling ball radius of 50 pixels, a light background, and a sliding paraboloid.

For the observation of CNFs, a JEOL JEM 1400 PLUS transmission electron microscopy (TEM) device (Tokyo, Japan) was utilized at the Spanish National Centre of Electronic Microscopy (CNME), following the methodology developed by Campano et al. (2020) [39]. The TEM microscope operated at 100 kV accelerating voltage, with an Orius SC200 CCD camera manufactured by Gatan (Pleasanton, CA, USA), featuring a resolution of 2048 × 2048 pixels and a pixel size of 7.4 microns.

With the TEM images obtained, projected fractal dimension (D2) was measured. D2 gives an idea of the “tree structure” of the sample, which can influence the mechanical properties of the material [40]. The acquired images were also processed and analyzed with ImageJ software, editing images to achieve good definition and high contrast in CNFs and converting these into 8-bit images. They were binarized through a threshold and corrected with a close filter. Elements in the images were selected individually and copied into a separate file. The fractal analysis was performed with the fractal box count plugin. To reduce processing times, this procedure was automatized through a script [41,42].

## 3. Results and Discussion

### 3.1. Pretreatment Effects on Fiber Morphology and Carboxyl Content of the Pulp

Table 4 shows the effect of the different pretreatments on the chemical composition of the pulp. The washing stage followed the enzymatic pretreatments to remove part of the ashes, which explains the reduction in ash content. This reduction in ashes could not be detected by Sanchez-Salvador et al. (2022) because of the low ash content of the virgin pulps [27]. Park et al. (2022) observed that ash in recycled paper has a high affinity for cellulase enzymes and attached to them, reducing enzyme efficiency [43]. Thus, ash attached to the enzyme is removed during pulp washing. In the case of TEMPO pretreatments, the increase in ash content is attributed to the production of NaCl as byproduct during the oxidation process and the addition of NaBr, both of which are not completely removed during washing [44]. Part of the extractives could be degraded or solubilized by the enzymes, as shown in Table 4. Consequently, this led to an increase in the percentage of cellulose. However, excessive amounts of enzymes or NaClO, particularly at a high TEMPO-mediated oxidation degree (TEMPO_15), could result in the excessive degradation of cellulose chains, leading to the formation of soluble oligosaccharides or sugars, which were subsequently removed during the washing stage. Hemicellulose seems to remain intact after the pretreatment process. 

**Figure 1:**
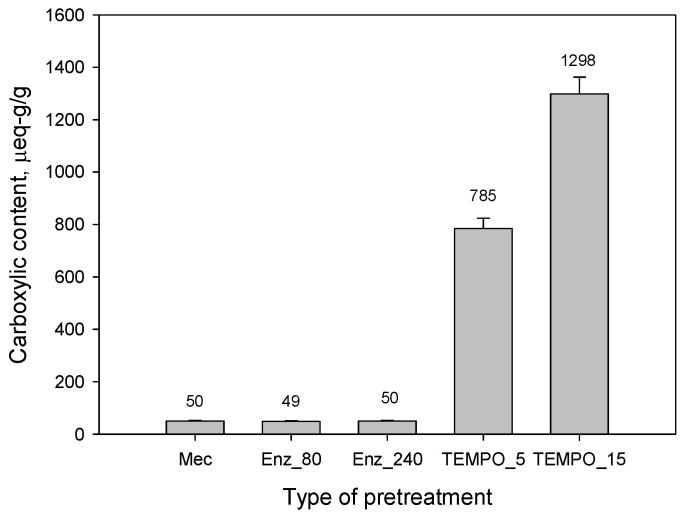
Carboxylic content of the recycled fibers after each pretreatment.

Figure 1 shows the carboxyl content of the pretreated pulps. The type of pretreatment and its severity determine the final quantity of carboxyl groups. Mechanical and enzymatic pretreatments do not contribute to the generation of carboxyl groups. In contrast, the number of carboxyl groups generated through TEMPO-mediated oxidation increases with the dosage of the oxidant, although not to the same extent as the NaClO dosage. This discrepancy arises because a portion of the NaClO is consumed in the oxidation of other components found in recovered papers, such as dissolved lignin and colloidal material [45]. Therefore, only a fraction of the NaClO is available to oxidize hydroxyl groups to carboxyl groups. Furthermore, there is a saturation phenomenon; when the dosage of NaClO surpasses a threshold, the overdosed NaClO remains in the medium and causes secondary reactions instead of effectively oxidizing cellulose. 

**Figure 2:**
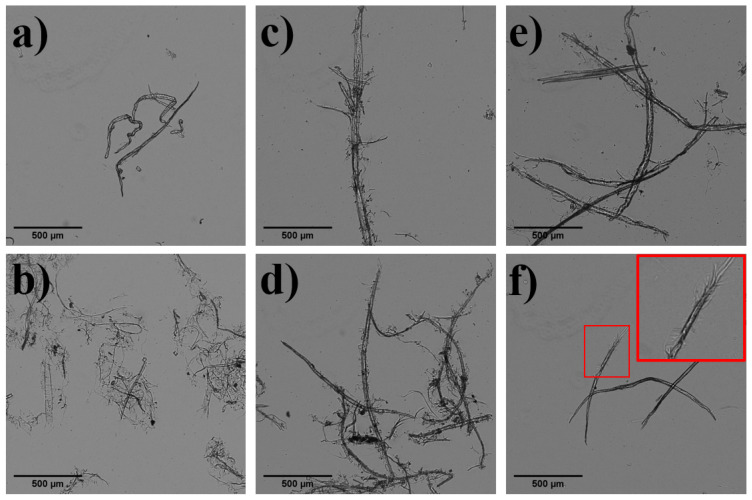
Optical images of the initial recycled pulp without pretreatment (**a**) and the pulps after different pretreatments: mechanical pretreatment (refining) (**b**); enzymatic hydrolysis with 80 mg/kg (**c**); enzymatic hydrolysis with 240 mg/kg (**d**); TEMPO-mediated oxidation with 5 NaClO/g (**e**); and TEMPO-mediated oxidation with 15 mmol NaClO/g (**f**).

Table 5 and Figure 2 present the morphological parameters of the fibers and the optical images of the pretreated fibers produced by different pretreatments. Table 5 shows fibers and fines which are detectable by the MorFi analyzer, whose detection limit is around 5 µm (for length). 

Figure 2 shows the optical images of the original and pretreated pulps. The original fibers (Figure 2a) exhibited a smooth surface, without notable external fibrillation. In contrast, the Mec pulp image (Figure 2b) shows an extremely high degree of fibrillation, which favors fine-and-fiber interaction, as well as the formation of smaller networks. During mechanical refining, fiber hornification is reversed since fibers recover part of their swelling ability, which increases their coarseness [46]. Fibrillation was also observed in the Enz_80- and Enz_240-pretreated pulps, albeit to a lesser extent (Figure 2c,d). This fact was also observed by other authors [27], so enzymes have been proposed as an alternative to refining to reduce the hornification effect. It is noticeable that Enz_240 induced greater fibrillation compared to Enz_80, resulting in a reduction in fiber length (Table 5). In contrast, TEMPO-mediated oxidation led to the lowest external fibrillation, as this reaction specifically oxidizes the cellulose chains, conferring electrostatic repulsive forces on interfibrils that reduce the energy required for nanofibrillation (Figure 2d,e), but without changing the fiber morphology during the chemical treatment. However, at high doses of NaClO, some external microfibrillation on the fibers can be observed due to the high electrostatic repulsive forces among chains due to the generated carboxyl groups in combination with hydrodynamic forces during pulp washing. This produces some CNFs from the smallest fines, as shown by the clean background of the optical image in Figure 2f. These CNFs are not visible with an optical microscope. Furthermore, some degradation of the amorphous part of the fibers by side-reactions can contribute to the external fibrillation (Figure 2f), which is in accord with the above. 

Furthermore, the TEMPO-mediated oxidation process resulted in the production of a significant quantity of fines and microfibers that decreased the values of coarseness and diameter. Some of the microfibers were too small to be clearly observed with an optical microscope and appeared as dots in Figure 2e. The optical images of TEMPO-mediated oxidized pulps reveal the treated fibers retain their structure prior to undergoing mechanical nanofibrillation. However, these images do not show the electrostatic repulsive forces occurring within the fibers, which are responsible for inducing nanofibrillation when shearing forces are applied.

### 3.2. Effect of the Pretreatment on the Properties of the Cellulose Nanofibers

Table 6 shows the results of the quantitative characterization of different CNFs produced using the highest intensity of HPH that corresponds to Sequence 5, as indicated in Table 3. GP was used to determine the aspect ratio of the fibers using crowding number (CN) theory [37,38,47].

**Figure 3:**
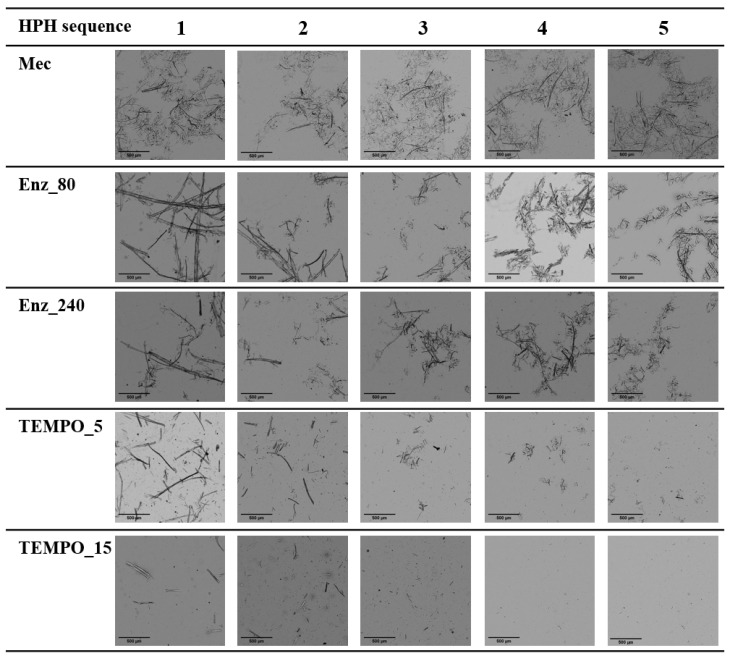
Optical microscopy images of CNFs (scale bar: 500 μm).

**Figure 4:**
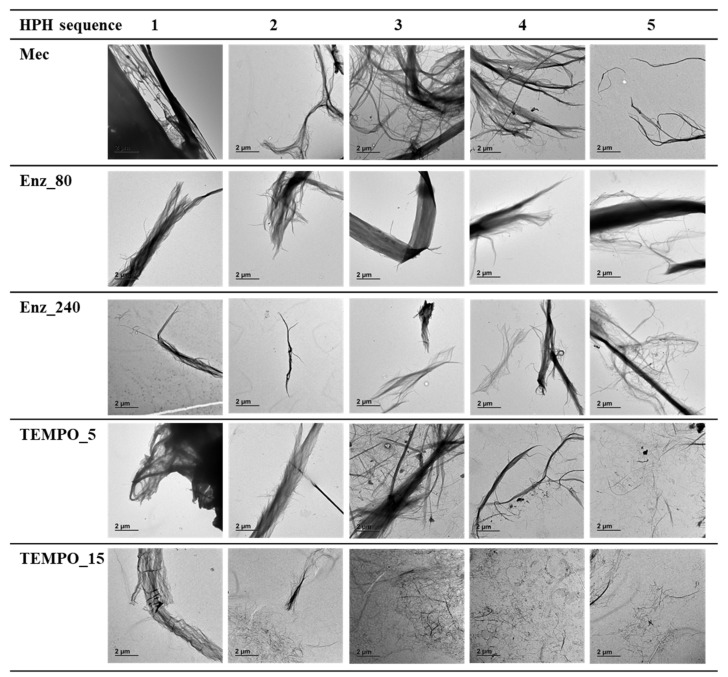
TEM images of CNFs (scale bar: 2 μm).

The aspect ratio of the nanofibers in the obtained hydrogels depends on the pulp pretreatment. The mechanically pretreated fibers (refined pulp) showed the highest aspect ratio, approximately 173. Figure 3 and Figure 4 provide visual qualitative evidence of the significantly high aspect ratio of this pretreated pulp compared to the others. They are optical (Figure 3) and TEM micrographs (Figure 4) of the twenty-five CNFs produced. Each CNF produced was analyzed by capturing twenty images, and each image in Figure 3 and Figure 4 was selected as a representative from those twenty images.

**Figure 5:**
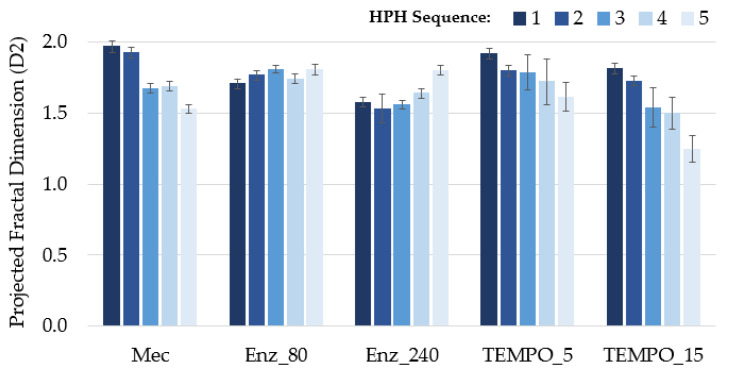
Projected fractal dimension (D2) calculated from TEM images at different HPH sequences.

Figure 3 and Figure 4 display, in the case of CNFs obtained by refining (Mec), the presence of highly fibrillated fibers longer than 500 µm, with a structure thinner than those obtained through enzymatic degradation followed by HPH. Optical images provide a higher visual field, but with lower resolution than TEM images. For example, the fluff shown in Figure 3 must be interpreted with the information given by TEM images, which show the level of nanofibrillation achieved. Figure 5 shows the D2 of refining pretreated pulp at different HPH sequences compared to the other pretreatments. Under soft HPH, it is possible to observe a high D2 value, near 2.0, due to the fibrils joining to the cellulose backbone, forming bundles. Increasing the intensity of homogenization, a decrease is observed in the D2, associated with fibrils separation from the main structure.

**Figure 6:**
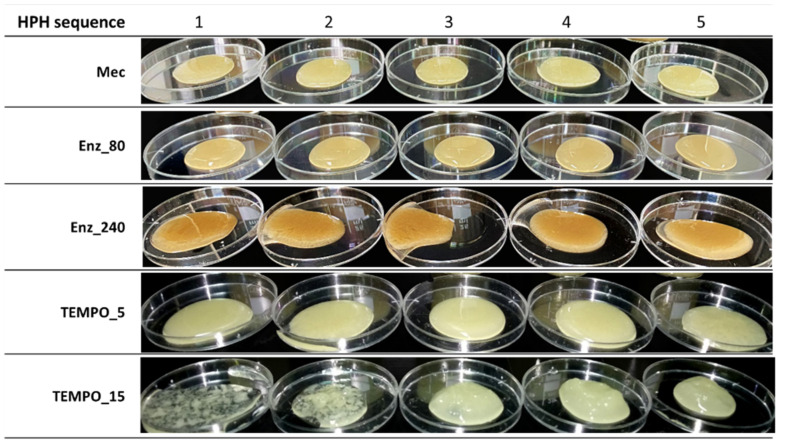
Photographs of obtained CNF hydrogels.

Both enzymatic hydrolysis and TEMPO-mediated oxidation pretreatments result in a decrease in fiber length and, consequently, aspect ratio compared to mechanically treated pulp. Enz_80 CNF suspension contains a substantial amount of short and thick fibers as D2 shows in Figure 5, with a lower value than for Mec. Figure 3 shows the length and Figure 4 shows the higher thickness of fibers that remained after the HPH process. A more intense enzymatic pretreatment, Enz_240, reduces the thickness, as evidenced by Figure 4, and the amount of these fibers that remain after HPH, as shown by the yield and transmittance values (Table 6), resulting in increased aspect ratio values. This effect on thickness is not observed in pretreated pulp, because it is the result of the combined effects of hydrolysis and HPH. The internal nanofibrillation caused by the enzyme favors the deconstruction of fiber in nanofibers during HPH. This effect is also observed in Figure 5, in which the D2 increases with the severity of HPH, which indicates the formation of microfibrils around the main structures with the main treatment. On the other hand, the orange color of Enz-80 and Enz-240 CNFs in Figure 6, which is even darker than Mec CNFs, could be due to the degradation products from enzymatic hydrolysis, which can produce oligosaccharides from the amorphous part of the cellulose chains decreasing the polymerization degree of cellulose [48].

Thus, the aspect ratio of TEMPO CNFs is notably lower than the others and is decreased with the NaClO dose. Figure 3 shows that only a few microfibrils in TEMPO are detected by optical microscopy after HPH (shown as small dots) and these become fewer and fewer as the intensity of homogenization increases, as D2 indicates with its decrease due to fiber individualization. However, Figure 4 shows a CNF network being responsible for forming a gel, as evidenced by the photograph in Figure 6 for TEMPO-15. The fact that none of the obtained nanocelluloses were transparent indicates that all of them have some microfibrils or nanocellulose aggregates that dispersed visible light. However, Figure 6 shows, the opacity of the gel suspension was lower in the case of TEMPO-15, as expected from the lower number of microfibers and higher amount of nanofibrillated cellulose and nanocrystals. The thin and short nanofibers observed in Figure 4 for TEMPO-15-CNF-5 account for the low aspect ratio values of these CNFs (Table 6). The sensitivity of cellulose to mechanical forces during the HPH process was increased by the cellulose oxidation and the removal of lignin due to the effect of NaClO during TEMPO-mediated oxidation. The soft yellow color of TEMPO-pretreated CNFs indicates lignin removal during the treatment. In fact, at the first stage of the reaction, NaClO is mainly consumed in lignin oxidation [45]. As a result, an intense HPH treatment caused the highest cutting effect, which accounts for the lowest aspect ratio (around 7) obtained for TEMPO_15-CNF-5, suggesting that the sufficiently intense TEMPO-mediated oxidation followed by an intense HPH process produces cellulose nanocrystals (CNCs), which have lower aspect ratios than CNFs [42]. According to Serra-Parareda et al. 2021, CNCs have D2 values under 1.4–1.5, whereas CNFs have higher D2 [42]. With this criterion, as Figure 5 shows, TEMPO_15-CNF-5 is in the CNC scale while TEMPO_15-CNF-3 and TEMPO_15-CNF-4 range from CNCs to CNFs. 

### 3.3. Effect of Homogenization Sequence on the Properties and Morphology of Cellulose Nanofibers

The homogenization pressure and cycles were varied in this study for the isolation of different CNF qualities. The final goal is to optimize the production of CNFs based on the desired morphology and properties for specific applications in the future. 

Figure 3 and Figure 4 show the significant influence of pretreatment on the nanofibrillation process of the pulps under different HPH intensities. The Mec pulp suffered minimal changes during HPH process, except for some internal fibrillation as shown in Figure 4. Although the fiber wall structure after enzymatic treatments exhibited limited signs of external fibrillation, this fibrillation noticeably increased with the intensity of subsequent mechanical treatment using HPH (Figure 3). Furthermore, Figure 4 shows some internal fibrillation. The most significant impact of HPH on morphology was observed in TEMPO_5 and TEMPO_15 CNFs. In both cases, the number of visible elements (Figure 3) decreased with increasing HPH intensity when observed through optical microscopy. This reduction was attributed to the intense internal nanofibrillation resulting from the combination of shearing forces and electrostatic repulsive forces. 

**Figure 7:**
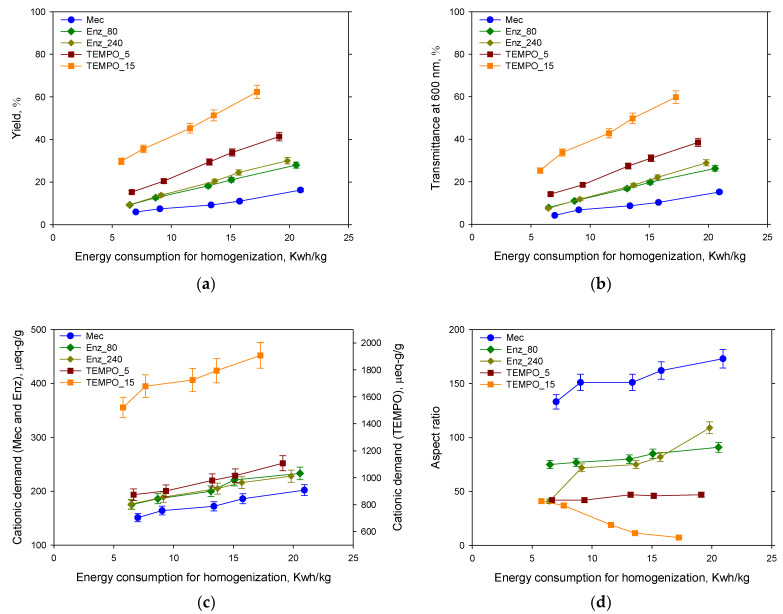
Nanofibrillation yield (**a**), transmittance (**b**) cationic demand (**c**) and aspect ratio (**d**) of the different CNF suspensions.

Figure 7 shows how the pretreatment determines the potential of HPH to enhance the properties of CNFs. In all cases, CNFs produced solely through mechanical pretreatment and HPH exhibited the lowest values of yield, transmittance, and CD, but the highest values of aspect ratio, measured by GP. Mechanical pretreatment caused external fibrillation and increased the accessibility of cellulose for subsequent treatments. However, it had limited impact on the yield and transmittance of CNFs. The energy consumption for each HPH sequence depends on the applied pretreatment. Enzymatic hydrolysis and TEMPO-mediated oxidation are more efficient in reducing energy consumption for homogenization as shown at Figure 7. The most efficient was TEMPO_15, as expected from the high anionic groups introduced on the fibers that generate electrostatic repulsive forces among cellulose chains helping nanofibrillation. Nanofibrillation yield and transmittance increased notably with energy consumption and with NaClO used in TEMPO-mediated oxidation. However, differences caused for the enzyme dose were not significant except for aspect ratio. This could indicate that the effect of the enzyme is limited by other variables, for example, due to poor accessibility of the enzyme to the cellulose chains due to the lower swelling ability and the presence of lignin. Figure 6 shows that Enz_80 and Enz_240 CNFs have different behavior with water. The latter tends to release water when it is deposited in a Petri dish. The interaction with water is key for GP determination because it affects the settling process. This differs from the results obtained for virgin pulps by Sanchez-Salvador et al. (2022) who observed a notable increase in transmittance, yield, and cationic demand by increasing the dose of enzyme threefold [27]. This evidences the effect of raw material composition in CNF production by enzymatic hydrolysis.

The CD of CNFs is not solely attributed to the presence of anionic groups on the nanofibril surface, but it is also influenced by the surface area available for PDADMAC adsorption, which depends on the external specific surface of the CNFs. This is evident from the increase in CD with the intensity of HPH treatment and with the level of fibrillation. However, the main factor affecting CD is the pretreatment itself. It is not possible to achieve a high CD for TEMPO CNFs with the studied combinations of pretreatment followed by HPH, due to the substantial number of anionic charges generated by TEMPO-mediated oxidation. TEMPO CNFs also exhibited higher yield and transmittance compared to others.

However, similar yields and transmittance to TEMPO_5 CNFs can be obtained using enzymatic hydrolysis followed by a more intense HPH treatment but with around 50% higher energy consumption. Even mechanical refining of the recycled pulp followed by the most intense HPH studied can produce CNFs of the same order of magnitude in terms of yield and transmittance, although with a lower value than the less fibrillated TEMPO_5 CNF. These findings differ from previous studies on Aspen pulp [28], where TEMPO-mediated oxidation followed by highly intense HPH enabled a yield of 100% and a transmittance around 95%, which were significantly higher than the values obtained with recovered paper. In this case, pulp composition is different due to the higher percentage of ashes and the presence of hornified fibers, which affects their capacity for being fibrillated and their interaction with water and chemicals and some dissolved and colloidal material.

The effect of HPH on aspect ratio is clearly influenced by the pretreatment. In the case of a mechanical pretreatment, the aspect ratio of CNFs increased with the energy consumption of HPH. A small effect on aspect ratio was observed for enzymatic pretreatments and a negligible effect was obtained in the case of TEMPO_5. However, the aspect ratio decreased with HPH intensity for TEMPO_15 CNF. These CNFs formed a hydrogel whose consistency increased with HPH intensity, which indicates that there was a 3D network of CNFs interacting with water. If the real aspect ratio of all the CNFs in TEMPO_15 was as low as that shown in Table 6 and Figure 7, the formation of a network would be unlikely. The aspect ratio decreased (Figure 7) while the consistency of the gel increased (Figure 6). This is due to the presence of non-settled particles during the sedimentation experiments. To better measure the aspect ratio, different, very diluted suspensions were prepared and the peak of sediment was measured after a long time (at the end of sedimentation process). In the case of TEMPO_15 CNF, there were some very stable nanoparticles in the suspension that did not settle even after several weeks, and the final aspect ratio was not real since it corresponded to the settleable material. The increase in HPH intensity increased the degree of nanofibrillation and CD values, which implies that a higher anionic charge is available to generate repulsive forces among particles, which can interact more strongly with water, forming a stable gel. 

## 4. Conclusions

By carefully selecting an appropriate pretreatment and level of severity, as well as an optimal HPH intensity, different CNF qualities can be produced from recovered paperboard. Based on the pretreatment and HPH intensity, the results can be used to predict the expected quality of CNFs. Recycled paperboard pulp allows one to obtain cost-effective colored and turbid hydrogels with a notable nanofibrillation yield (63%) and transmittance (60%), which can be used as reinforcing aids in board-recycling mills. The hornification of the fibers and the presence of ashes and lignin affects the efficiency of enzymatic hydrolysis, with a dose of 80 mg/g of pulp being enough to obtain the maximal performance of the produced CNF. The characteristics of the produced CNFs and the impact of HPH on nanofibrillation are both significantly influenced by the pulp pretreatment. This enables one to choose the best production strategy with the highest yield in terms of produced CNF for the required CNF quality in various applications.

## Figures and Tables

**Table 1:** Chemical composition of recycled paperboard pulp.

Compound	Percentage (wt%)
Cellulose	56.8 ± 0.4
Hemicelluloses	14.0 ± 0.2
Soluble lignin	4.6 ± 0.2
Klason lignin	11.6 ± 0.2
Extractives	1.7 ± 0.1
Ashes	11.3 ± 0.4

**Table 2:** Pretreatments studied to produce CNFs from recycled paperboard.

Pretreatment Identification	Description	Intensity	Conditions
Mec	Refining at PFI refiner ISO standard 5264-2 (2011)	20,000 revolutions	PC = 10% 25 °C
Enz_80	Enzymatic hydrolysis Denaturalization (80 °C, 15 min) Pulp washing	Dose of enzyme: 80 mg/kg	PC = 5% pH = 7 Time = 4 h T = 50 °C
Enz_240		240 mg/kg	
TEMPO_5	TEMPO-mediated oxidation Pulp washing with water and filtration up to pH = 7	5 mol NaClO/kg	PC = 1% pH = 10 T = 25 °C
TEMPO_15		15 mol NaClO/kg	

All the doses are expressed as dry agent per kg of dry pulp. PC: Pulp consistency.

**Table 3:** HPH conditions to produce CNFs.

HPH Sequence Number	Number of Cycles		
	300 bar	600 bar	900 bar
1	3	0	0
2	3	1	0
3	3	3	0
4	3	3	1
5	3	3	3

**Table 4:** Composition of pretreated pulps.

	Extractives	Ashes	Soluble Lignin	Klason Lignin	Cellulose	Hemi- Cellulose
Mec	1.8 ± 0.1	12.6 ± 0.3	4.3 ± 0.2	12.5 ± 0.2	56.4 ± 0.4	12.5 ± 0.2
Enz_80	1.1 ± 0.1	7.4 ± 0.2	4.5 ± 0.2	12.5 ± 0.2	60.6 ± 0.4	13.8 ± 0.2
Enz_240	1.0 ± 0.1	8.1 ± 0.2	4.9 ± 0.2	15.6 ± 0.2	54.8 ± 0.4	15.3 ± 0.2
TEMPO_5	2.0 ± 0.2	12.2 ± 0.2	9.5 ± 1.6	7.5 ± 0.5	55.1 ± 1.6	13.7 ± 0.4
TEMPO_15	1.9 ± 0.2	16.2 ± 0.3	10.5 ± 1.4	3.2 ± 0.2	45.5 ± 1.1	22.7 ± 0.5

**Table 5:** Morphological characteristics of the pulps pretreated with refining, enzymatic hydrolysis, and TEMPO-mediated oxidation prior to the HPH.

Pretreatment	Length_w_ (μm)	Diameter (μm)	Coarseness (mg/m)	Fines (%)
Untreated pulp	881 ± 18	24.6 ± 0.1	0.155 ± 0.0003	37.9 ± 2.1
Mec	715 ± 95	22.3 ± 0.6	0.156 ± 0.011	47.2 ± 7.5
Enz_80	541 ± 22	23.9 ± 1.3	0.144 ± 0.001	38.3 ± 1.1
Enz_240	351 ± 34	23.7 ± 1.7	0.098 ± 0.020	53.6 ± 4.0
TEMPO_5	258 ± 11	20.0 ± 1.0	0.046 ± 0.017	51.2 ± 4.6
TEMPO_15	249 ± 11	18.1 ± 0.7	0.037 ± 0.012	61.0 ± 3.9

Length_w_: length weighted in length.

**Table 6:** Properties of cellulose nanofibers produced by different pretreatments and followed by Sequence 5 HPH (3 passes at 300 bar + 3passes at 600 bar + 3 passes at 900 bar).

	Yield (%)	Transmittance at 600 nm (%)	CD (μeq-g/g)	Aspect Ratio (GP)
Mec	16.2	15.2	202	173
Enz_80	28.0	26.3	233	91
Enz_240	30.0	28.9	228	109
TEMPO_5	41.4	38.5	1108	47
TEMPO_15	62.3	59.8	1908	7.3

## Data Availability

Data can be made available upon request to the corresponding author.
